# Predicting cardiometabolic disease in medical students using FibroScan and 30-year Framingham risk scores

**DOI:** 10.3389/fmed.2024.1431935

**Published:** 2024-09-26

**Authors:** Charu Sharma, Muhammad Jawad Hashim, Javed Yasin, Mahra Rashid Salim Alnaqbi, Abdulla Saeed Ahmed Alkaabi, Mohammed Saif Mohammed Aldhaheri, Juma Alkaabi, Adnan Agha

**Affiliations:** ^1^Department of Internal Medicine, College of Medicine and Health Sciences, United Arab Emirates University, Al Ain, United Arab Emirates; ^2^Department of Family Medicine, College of Medicine and Health Sciences, United Arab Emirates University, Al Ain, United Arab Emirates

**Keywords:** cardiovascular disease, cardiovascular risk prediction, metabolic dysfunction-associated steatotic liver disease, Framingham risk score, FibroScan

## Abstract

**Introduction:**

Metabolic dysfunction-associated steatotic liver disease (MASLD) has become a major cause of end-stage hepatic disease worldwide requiring liver transplantation, whereas cardiovascular disease (CVD) remains the leading cause of morbidity and mortality globally. Development of MASLD and CVD among young adults is understudied. This study aimed to assess CVD risk in healthy young medical university students using lipid-based and body mass index (BMI)-based 30-year Framingham risk scores (FS30) and to evaluate disease burden for asymptomatic patients with MASLD by performing FibroScan.

**Methods:**

We included medical university students aged 18–30 years without any known medical conditions. All participants underwent physical and anthropometric measurements, and completed a questionnaire. Blood samples were collected for the analysis of glycosylated haemoglobin levels, renal and liver function, biomarker analysis to calculate liver fibrosis risk, and subclinical atherosclerosis biomarkers. Liver stiffness measurements (LSM) and controlled attenuation parameter (CAP) values were measured using FibroScan 430 mini to calculate liver fibrosis and steatosis, respectively. FS30 based on body mass index (FS30-BMI) and lipid levels (FS30-Lipid) were also calculated.

**Results:**

Overall, 138 medical students participated in this study after providing informed consent. Using FS30-Lipid and FS30-BMI, CVD risk was identified in two (1.5%; *n* = 138) and 23 (17.6%; *n* = 132) individuals, respectively. MASLD fibrosis was identified based on FibroScan LSMs >7.0 kPa in 12 medical students (9.4%, *n* = 128; 95% CI, 4.7–14.8%). Consumption of coffee and sugary soft drinks were predictive of liver fibrosis. In total, 36 students (28.6%; *n* = 128) were found to have hepatic steatosis based on FibroScan CAP values >236 dB, and the predictive factors included increased body fat percentage, male sex, and lack of physical activity. Levels of inflammatory biomarkers, such as C-reactive protein and lipids were not elevated in participants with MASLD.

**Discussion:**

CVD risk was identified in >17% of young medical students. The frequency of liver fibrosis and steatosis was also high among the participants, indicating that liver damage starts at a relatively early age. Early intervention is needed among young adults via health promotion and lifestyle changes.

## 1 Introduction

Cardiovascular disease (CVD) is the leading cause of morbidity and mortality worldwide and almost 90% of deaths are secondary to ischemic heart disease and stroke, with the commonest risk factors being diabetes mellitus, obesity, dyslipidaemia, and high-blood pressure (1). The global prevalence of diabetes is only 9.3%. In the United Arab Emirates (UAE), it is present in nearly twice as much (15.4%) in the adult population and another significant proportion of patients with diabetes remain undiagnosed (2). These CVD risk factors can accumulate, starting in adolescence and continuing into middle- and old-age, with increased risk of atherosclerosis and CVD development over time (3, 4). Other than being a significant cardiovascular risk factor, both diabetes and obesity can also cause metabolic dysfunction-associated steatotic liver disease (MASLD), formerly known as nonalcoholic fatty liver disease (NAFLD), which has become the leading reason for liver transplantation in the West (5). Various non-invasive methods that can detect the presence of advanced fibrosis in MASLD cases are available, including clinical or biochemical scoring systems, such as NAFLD fibrosis scores (NFS), fibrosis 4 index (FIB-4), and imaging techniques, including magnetic resonance elastography (6).

The global prevalence of MASLD is reported to be 32% among adults, and higher among men (40%), compared with women (26%). The prevalence of MASLD varies substantially by region across the world, contributed by differing rates of obesity, and genetic and socioeconomic factors (5). The prevalence of MASLD in Asia is 30% (7), whereas that in Europe is 30.9% (7, 8) and in UAE is 25% as in 2017; this is projected to increase to 46% by 2030 (9). A small number of studies have reported on cardiometabolic risk and MASLD among lean and non-lean individuals (10), and on metabolically healthy to metabolically unhealthy participants from baseline to 10 years later (11); however, there is no evidence reported in the literature which can predict the 30-year cardiometabolic risk as well as presence or risk of MASLD and diabetes in healthy young university students. In the UAE, the recently concluded healthy future study showed that cardiometabolic risk factors are highly prevalent in the population of Emirati ethnicity and can contribute to increasing the burden of CVD risk (12). Cardiometabolic risk factors can be assessed using measurement of physical and anthropometric indices, which can also serve as a useful tool to identify future risk of developing obesity (13). However, recent studies have shown that subclinical atherosclerosis may exist even without these traditional cardiometabolic risk factors and new biomarkers, such as apolipoprotein A (ApoA), Apolipoprotein B (Apo B), polymeric immunoglobulin receptor (PIGR), immunoglobulin heavy constant alpha 2 (IGHA2), and heparin cofactor 2 (HEP2) can predict subclinical atherosclerosis independently and offer potential for future disease prediction (14).

Longer-term risk assessments are known to be better predictors of subclinical and clinical CVD than are shorter-term risk predictions. They account for competing causes of death, thereby providing a more realistic assessment of the overall CVD burden (15). The currently used evidence-based traditional risk score usually predicts 10-year risk of cardiovascular events and includes tools such as systematic coronary risk evaluation, Framingham risk score (10 years), assign risk score, Reynold risk score, and prospective cardiovascular Münster risk score, among others (16). A long term cardiovascular risk score, 30-year Framingham heart study score (FS30), based on Framingham cardiovascular study cohort of individuals between the ages of 20–59 years has been proposed, and this model has shown excellent performance with cross-validated discrimination of C = 0.803 and calibration of χ^2^ = 4.25 (*p* = 0.894) (17). The FS30 is the only longer-term risk prediction function designed to be used with young adults because it accurately estimates the extent of CVD risk and future disease burden in this population. There is CVD risk assessment studies based on FS30 in the region.

In this study, we sought to fill this knowledge gap by primarily assessing the 30-year risk for future cardiovascular events using available predictive scores, such as FS30, and assessing the presence of subclinical atherosclerosis using available serum biomarkers in young university students. We also aimed to estimate the presence of undiagnosed diabetes, or other cardiovascular risk factors based on biomarkers, anthropometric and physical measurements, as well as identify MASLD cases using FibroScan, a non-invasive bedside tool. Finally, we aimed to assess whether non-invasive clinic-based tools could accurately predict the presence of MASLD.

## 2 Materials and methods

### 2.1 Study population

We conducted a prospective cross-sectional study with participating students (after informed consent) at the UAE University Al-Ain. We planned to include a minimum of 112 participants as per study power calculations with an estimated prevalence of 37% of hepatic steatosis (power 80%, and alpha level 5%). The study included university students aged 18 to 30 years without any medical condition that caused them to have a disability that limited their activities of daily living and those who agreed to participate via informed consent. The exclusion criteria were students with mental health issues on undergoing treatment, established cardiovascular disease or previous cardiovascular events, or having any history of alcohol use.

This study was conducted in accordance with the Declaration of Helsinki and was reviewed by our institutional ethics committee. The study was approved by UAE University Human Medical Research Ethics Committee (UAEU.HREC) with approval no# ERH_2023_2353. Informed consent were obtained from all participants involved in this study.

### 2.2 Clinical parameters

The participants completed a questionnaire which included questions regarding their lifestyle, diet, exercise, and other pertinent history, including family history of CVD, smoking, and stress. All participants underwent various physical and anthropometric measurements, including weight, height, blood pressure and body mass index (BMI). A tape measure was used for the determination of body indices. The three common measurements obtained were waist, hip, and neck circumferences, and the standard unit used was the SI unit of centimetre (cm).

### 2.3 Blood sampling

All students participating in the study fasted for at least 4 h, and blood samples were collected via venipuncture. Plasma was separated and stored at −20°C until analysis. The plasma samples were processed for biomarker analysis including low-density lipoprotein, high-density lipoprotein, triglycerides, total cholesterol, urea, creatinine, total protein, albumin, glycosylated haemoglobin (HbA1c), and renal and liver function tests for FIB-4 calculation. Currently recognised subclinical atherosclerotic biomarkers such as ApoA, ApoB, PIGR; IGHA2, and haptoglobin and HEP2 were also determined. Haptoglobin, blood glucose and HbA1c were analysed using an automated analyser, Integra 400 Plus (Roche Diagnostics, Mannheim, Germany). To measure human immunoglobulin A2 and human PIGR levels, we used a commercially available enzyme-linked immunosorbent assay kit from Elabscience Biotechnology Inc., United States.

### 2.4 Body fat measurement

Body fat thickness measurements were performed using the Harpenden Skinfold Calliper-RH15 (Baty International, England), which is a precise tool used to determine the thickness of skin fat in specific areas, such as the triceps and waist. The Calliper provided measurements in centimetre and captured increments of 0.2 mm. Participants also underwent body fat (BF) composition analysis, and electrical impedance or BF percentage using a TBF-300 Tanita Body composition analyser (Meano-Cho, Tokyo Japan). Tanita was used to determine body composition, including BF, BF mass (kg), fat-free mass, body muscle mass, and total body water.

### 2.5 MASLD assessment using FibroScan

The LSMs were conducted using FibroScan 430 Mini (Fibro Scan mini+430, France) with both M and XL probes. Measurements were performed following a 3–4 h of fasting by the medical students. The tip of the transducer was covered with a drop of gel and placed perpendicularly in the intercostal space of the participant, who was required to lie in the dorsal decubitus position with the right arm at maximal abduction. Scanning was conducted in a region encompassing the sixth, seventh, and eighth intercostal spaces between anterior axillary and mid-axillary lines. Vibration-controlled transient elastography utilising FibroScan provided the LSMs, which were expressed in kilopascals (kPa), whereas liver steatosis measurements were performed via controlled attenuation parameter (CAP) values expressed in decibels per m (dB/m). A cut-off value of 7 kPa was used to define elevated liver stiffness in the FibroScan results.

### 2.6 30-year cardiovascular risk assessment

For all participants, the FS30 based on BMI (FS30-BMI) and lipid levels (FS30-Lipid) were calculated using an online tool available^1^ cardiovascular-disease-30-year-risk/ developed by Pencina et al. (17). Although a 12% risk is generally considered elevated, with >40% considered as high risk, we used FS30 ≥ 6% as a cut-off for above-normal or elevated CVD risk (with <6% considered as no risk for CVD) as almost all students had values below the 12% threshold.

### 2.7 Markers for predicting MASLD

Liver function tests, including alanine aminotransferase (ALT), aspartate aminotransferase (AST), albumin and prothrombin clotting time, and other clinical or biochemical scoring systems, such as FIB-4 and NFS, were calculated and used to predict risk of MASLD. The abnormal cut-off scores for FIB-4 and NFS were taken as ≥1.45 and ≥ −1.455, respectively (18).

### 2.8 Statistical analysis

Statistical analysis was performed using SPSS v.22.0 (IBM SPSS Inc., Armonk NY). Data were collected using paper forms, entered into an Excel spreadsheet, and subsequently imported into SPSS for statistical analysis. After obtaining descriptive measures, bivariate analysis was conducted using Pearson correlations and one-way analysis of variance tests. Multivariate analysis was carried out with stepwise linear regression. An alpha level of 0.05 was considered statistically significant.

## 3 Results

A total of 138 medical students participated in the study. The participants were young adults with a mean age of 20.0 years (standard deviation [SD], 1.6; range: 17–24 years). A majority of the students were female (86 of 138 [62.3%]), reflecting the overall sex distribution in the medical college. Demographic characteristics of the participants are summarised in Table 1, which includes breakdown by sex. Among the 138 participants, 10 did not undergo the liver FibroScan. For an additional two participants, the LSM measurements were obtained; however, the CAP values could not be assessed due to technical issues. Six students did not complete their Tanita BF or body weight/BMI measurements; however, all of them underwent complete blood work-up. None of the students had any history of cardiovascular disease or any alcohol intake.

**Table 1:** Clinical characteristics of study participants (*n* = 138 except where indicated).

	Females N (%)	Males N (%)	All N (%)	*p*-value
**Age**				
≤20 years	52 (66.7)	26 (33.3)	78 (56.5)	NS
21+ years	34 (56.7)	26 (43.3)	60 (43.5)	
**BMI (kg/m** ^2^ **; *N* = 132)**				
Underweight <20	17 (21.3)	6 (11.5)	23 (17.4)	NS
Normal 20–24	31 (38.8)	19 (36.5)	50 (37.9)	
Overweight 25–29	23 (28.8)	14 (26.9)	37 (28.0)	
Obese 30–34	6 (7.5)	8 (15.4)	14 (10.6)	
Morbidly obese 35+	3 (3.8)	5 (9.6)	8 (6.1)	
Diabetes	2 (2.4)	0 (0)	2 (1.4)	NS
Hypertension	0 (0)	1 (1.9)	1 (0.7)	NS
Dyslipidemia	3 (3.5)	1 (1.9)	4 (2.9)	NS
Asthma	6 (7.1)	4 (7.7)	10 (7.2)	NS
Inhaler use	1 (1.2)	2 (3.8)	3 (2.2)	NS
Outside foods daily	22 (25.6)	29 (55.8)	51 (37.0)	< 0.001
Sugared drinks daily	23 (27.1)	28 (53.8)	51 (37.2)	0.002
Coffee 2 or more cups daily	26 (31.0)	14 (27.5)	40 (29.6)	NS
Sedentary	45 (52.9)	15 (28.8)	60 (43.8)	0.006
Stress/anxiety	14 (16.5)	2 (3.8)	16 (11.7)	0.026
Smoking	1 (1.2)	5 (9.6)	6 (4.4)	0.019
Family history of CVD	17 (20.0)	3 (5.8)	20 (14.6)	0.022

BMI, Body mass index; CVD, Cardiovascular disease; NS, Non-significant.

There were significant sex differences in the daily consumption of take-away food (*p* < 0.001), and sugary soft drinks (*p* = 0.002). Stress (*p* = 0.019) and familial history of CVD (*p* = 0.022) were significantly elevated among the female participants. We did not observe any differences in BMI among the participants.

### 3.1 CVD: FS30 risk assessment

As we wanted to assess the CVD risk, FS30-Lipid and FS30-BMI were calculated in terms of having a risk of hard cardiovascular (myocardial infarction, and fatal or non-fatal stroke) and full cardiovascular events (which included coronary insufficiency, angina pectoris, stroke plus transient ischemic attack, intermittent claudication, and congestive heart failure in addition to hard cardiovascular events). Based on the FS30-Lipid results, only 2 (1.5%) of participants were found to be at risk for full cardiovascular events. On calculating FS30-BMI, 23 (17.6%) participants were identified to be at risk for full cardiovascular events (CVE), with five (3.8%) of them having a risk of hard CVE (Table 2).

**Table 2:** FibroScan and Framingham risk scores of participants.

	Females N (%)	Males N (%)	All N (%)	*p*-value
Liver stiffness >7 kPa	5 (6.5)	7 (13.7)	12 (9.4)	NS
**Liver Steatosis (dB/m)**				
Absent (S0) <236	61 (81.3)	29 (56.9)	90 (71.4)	0.003
Mild (S1) 236–269	11 (14.7)	10 (19.6)	21 (16.7)	
Moderate (S2) 270–301	3 (4.0)	6 (11.8)	9 (7.1)	
Severe (S3) ≥302	0 (0)	6 (11.8)	6 (4.8)	
Fib-4 score > 1.45	0 (0)	1 (1.9)	1(0.7)	NS
FS No risk of Full CVD (FS30-lipid)	79 (60.3%)	50 (38.2%)	129 (98.5%)	
FS Risk of Full CVD (FS30-lipid)	0 (0%)	2 (1.5%)	2 (1.5%)	
FS No risk of hard CVD (FS30-lipid)	79 (60.3%)	52 (39.7%)	131 (100%)	
FS Risk of hard CVD (FS30-lipid)	0 (0%)	0 (0%)	0 (0%)	
FS No risk of Full CVD (FS30-BMI)	77 (58.8%)	31 (23.7%)	108 (82.4%)	
FS Risk of Full CVD (FS30-BMI)	2 (1.5%)	21 (16.0%)	23 (17.6%)	
FS No risk of hard CVD (FS30-BMI)	79 (60.3%)	47 (35.9%)	126 (96.2%)	
FS Risk of hard CVD (FS30-BMI)	0 (0%)	5 (3.8%)	5 (3.8%)	
FibroScan liver stiffness (kPa)	5 (6.5%)	7 (13.7)	12 (9.4%)	

There were 86 (62.3%) females and 52 (37.7%) males.

### 3.2 FibroScan results

To assess the liver fibrosis, LSMs were measured using FibroScan ranged from 2.50 to 13.7 kPa (mean 5.02; SD 1.75; *n* = 128; 10 missing values). Using an LSM of >7.0 kPa as a cut-off, 12 of 128 (9.4%; 95% CI, 4.7–14.8%) students had elevated readings (Table 2).

The LSMs were weakly correlated with steatosis (*r* = 0.20, *p* = 0.02) and age (*r* = 0.22, *p* = 0.01) but not with the levels of liver biomarkers, such as ALT, AST, and albumin.

The CAP values measured using FibroScan (which assesses hepatic fatty infiltration) were moderately correlated with BMI (*r* = 0.50, *p* < 0.001) and to a lesser extent with waist circumference (*r* = 0.48, *p* < 0.001) and body fat mass (*r* = 0.47, *p* < 0.001). The CAP values were also associated with higher risk of full CVE (*r* = 0.42, *p* < 0.001) using FS30-BMI and higher risk of hard CVE (*r* = 0.43, *p* < 0.001) using FS30-BMI (Table 3).

**Table 3:** Comparison of participants with and without elevated liver stiffness.

	Elevated liver stiffness Mean ± SD	Control Mean ± SD	*p*-value
FS Risk of Full CVD (FS30-lipid)	1.3 ± 1.4	1.1 ± 1.0	
FS Risk of Hard CVD (FS30-lipid)	0.1 ± 0.3	0.0 ± 0.4	
FS Risk of Full CVD (FS30-BMI)	4.1 ± 3.8	2.9 ± 2.6	
FS Risk of Hard CVD (FS30-BMI)	1.8 ± 2.1	1.3 ± 1.4	
FibroScan liver stiffness (kPa)	9.0 ± 2.2	4.6 ± 1.0	
WBC (10^3^/mm^3^)	7.7 ± 2.6	6.4 ± 2.2	0.048
RBC (10^6^/mm^3^)	6.1 ± 0.7	5.7 ± 0.9	
Haemoglobin (g/dl)	17.3 ± 2.2	15.6 ± 2.5	0.021
Haematocrit (%)	49.4 ± 6.1	45.2 ± 7.1	0.050
Platelets (10^3^/mm^3^)	226.4 ± 59.6	250.7 ± 76.8	
MCV (fl)	80.6 ± 6.7	79.3 ± 8.2	
MCH (pg)	28.3 ± 3.0	27.4 ± 3.6	
MCHC (g/dl)	35.1 ± 1.2	34.4 ± 1.2	
HbA1c (%)	5.1 ± 0.4	5.2 ± 0.5	
Plasma glucose (mmol/L)	5.3 ± 0.6	5.6 ± 1.0	
Albumin (g/L)	52.2 ± 2.9	52.0 ± 3.8	
Total protein (g/L)	70.8 ± 5.0	70.7 ± 5.1	
ALT (U/L)	19.8 ± 8.1	17.7 ± 5.9	
AST (U/L)	27.0 ± 8.6	22.6 ± 7.7	
Haptoglobin (g/L)	1.1 ± 0.5	1.3 ± 0.5	
Apo-A (g/L)	1.7 ± 0.3	1.8 ± 0.3	
Apo-B (g/L)	1.0 ± 0.3	1.0 ± 0.2	
TC (mmol/L)	4.5 ± 0.8	4.7 ± 0.8	
HDL (mmol/L)	1.5 ± 0.4	1.6 ± 0.4	
LDL (mmol/L)	2.9 ± 0.7	2.9 ± 0.7	
TG (mmol/L)	0.8 ± 0.3	0.9 ± 0.5	
BUN (mg/dl)	8.5 ± 2.9	7.7 ± 2.0	
Creatinine (umol/l)	65.3 ± 14.2	64.8 ± 12.5	
Urea (mmol/L)	3.0 ± 1.1	2.7 ± 0.7	
hs-CRP (mg/l)	1.5 ± 2.2	2.4 ± 3.7	
pIgR (pg/ml)	704.4 ± 418.9	755.9 ± 423.2	
IgA2 (g/L)	0.3 ± 0.1	0.3 ± 0.1	

FS, Framingham Score; MCV, mean corpuscular volume; MCH, mean corpuscular haemoglobin; MCHC, mean corpuscular haemoglobin concentration; HbA1c, Haemoglobin A1c; ALT, Alanine Aminotransferase; AST, Aspartate Aminotransferase; Apo-A, Apolipoprotein-A; Apo-B, Apolipoprotein-B; TC, total cholesterol; HDL, high-density lipoprotein, LDL, low-density lipoprotein, TG, triglycerides; BUN, blood urea nitrogen; hs-CRP, high sensitivity C-Reactive protein; pIgR, polymeric immunoglobulin receptor, IgA2, Immunoglobulins A2.

### 3.3 Fib-4

FIB-4 was used biochemical predictor for liver fibrosis. There was only one participant (male) with an elevated FIB-4 score of 1.59, while all female participants did not have an elevated FIB-4 score of >1.45. FIB-4 correlated with AST (*r* = 0.57, *p* < 0.001) and weakly correlated with age (*r* = 0.27, *p* = 0.001) and ALT (*r* = 0.27, *p* = 0.001) but not with other parameters such as LSMs and CAP values, and FS for major (hard) and all (full) cardiovascular events obtained using either lipid or BMI metrics, and anthropometric measurements.

A FIB-4 cut-off value of 1.45 was not useful in discriminating between high and low liver stiffness (mean FibroScan values of 5.03 and 5.40 kPa, respectively; *p* = 0.833).

### 3.4 Cardiovascular risk

CVD risk was assessed by assessing Framingham risk scores and these were analysed for correlations with MASLD parameters.

The risk for all or [or full cardiovascular events (CVE)] by FS30-Lipid was associated with higher CAP value (*r* = 0.28, *p* = 0.001), age (*r* = 0.33, *p* < 0.001), and body measurements, such as BMI (*r* = 0.41, *p* < 0.001), waist circumference (*r* = 0.46, *p* < 0.001), BF mass (*r* = 0.37, *p* < 0.001), and ALT (*r* = 0.33, *p* < 0 0.001) but not with LSM in kPa, or FIB-4.

The risk for hard CVE using lipid metrics (FS30-Lipid) did not correlate with any MASLD parameters, such as the LSM or CAP value, age, anthropometric measurements (BMI, BF percentage, and BF mass), or liver markers (ALT, AST, albumin; *r* < 0.5 for all correlations).

The risk for full CVE obtained using FS30-BMI was associated with CAP values (*r* = 0.42, *p* < 0.001) but not with LSM or FIB-4. This was also the case for the risk of hard CVE obtained using FS30-BMI and CAP values (*r* = 0.43, *p* < 0.001). As expected, the cardiovascular risk scores obtained using BMI-based calculations showed stronger correlations with BMI and waist circumference, compared with those obtained using lipid-based formulas.

There were strong correlations (*r* > 0.8, *p* < 0.001) among FS30 obtained using lipid- and BMI-based formulae (Table 4).

**Table 4:** Comparison of participants with and without liver steatosis (*n* = 138).

	Liver steatosis N (%)	Normal N (%)	All N (%)	*p*-value
**Age**				
≤20 years	12 (40.0)	56 (58.3)	68 (54.0)	0.079
21+ years	18 (60.0)	40 (41.7)	58 (46.0)	
Gender Female	10 (33.3)	65 (67.7)	75 (59.5)	< 0.001
Male	20 (66.7)	31 (32.3)	20 (66.7)	
**BMI (kg/m** ^2^ **)**				
Underweight <20	3 (10.0)	18 (18.8)	21 (16.7)	< 0.001
Normal 20–24	4 (13.3)	44 (45.8)	48 (38.1)	
Overweight 25–29	9 (30.0)	27 (28.1)	36 (28.6)	
Obese 30–34	9 (30.0)	5 (5.2)	14 (11.1)	
Morbidly obese 35+	5 (16.7)	2 (2.1)	7 (5.6)	
Diabetes	0 (0)	2 (2.1)	2 (1.6)	0.423
Hypertension	0 (0)	1 (1.1)	1 (1.8)	0.573
Dyslipidemia	1 (3.3)	3 (3.2)	4 (3.2)	0.962
Asthma	2 (6.7)	6 (6.3)	8 (6.4)	0.945
Inhaler use	1 (3.3)	2 (2.1)	3 (2.4)	0.702
Outside foods daily	11 (36.7)	35 (36.5)	46 (36.5)	0.983
Sugared drinks daily	12 (40.0)	32 (33.7)	44 (35.2)	0.528
Coffee 2 or more cups daily	7 (25.0)	30 (31.6)	37 (30.1)	0.505
Sedentary	15 (50.0)	39 (41.1)	54 (43.2)	0.388
Stress/anxiety	3 (10.0)	10 (10.5)	13 (10.4)	0.934
Smoking	3 (10.0)	3 (3.2)	6 (4.8)	0.126
Family history of CVD	5 (16.7)	14 (14.7)	19 (15.2)	0.797

### 3.5 Anthropometric measurements

We wanted assess the relationship of waist circumference, body fat and other anthropometric measurements with presence of MASLD. Waist circumference was strongly correlated with BMI (*r* = 0.81, *p* < 0.001). However, BF analysis metrics were not correlated with MASLD measurements, such as LSM or CAP values, or FIB-4. BF percentage was associated with BMI (*r* = 0.78, *p* < 0.001) and waist circumference (*r* = 0.54, *p* < 0.001).

### 3.6 Multivariate analyses

FibroScan liver stiffness (measured in kPa; a marker for MASLD) was predicted by ALT only predicted by ALT only, whereas the other parameters did not have any predictive value (adjusted R-squared = 0.03). A machine automated modelling procedure showed that AST, asthma, coffee intake, and the frequency of sugary soft drink consumption were predictive of LSM.

A higher CAP value was predicted by male sex, high BF percent, and a lack of regular physical activity but not by dyslipidaemia, diabetes, hypertension, smoking, inflammatory biomarkers, or liver biomarkers (124 cases included, adjusted R-squared = 0.38).

FIB-4 was predicted by male sex, higher age, and frequent consumption (daily intake) of take-away food but not by other parameters (adjusted R-squared = 0.20).

Multivariate models for FS showed better predictive power.

The risk of full cardiovascular events obtained using FS30-Lipid was predicted by hypertension, Apo B, sex, smoking, age, Apo A, BF percent (in order of decreasing influence, as indicated by standardised beta coefficients) but not by other parameters, such as MASLD markers including LSM, CAP and FIB-4 (adjusted R-squared = 0.83; Table 5).

**Table 5:** Predictors of liver disease and cardiovascular risk based on multivariate analyses.

Outcome	Predictors	Explanatory power
Liver stiffness	AST	Machine automated modelling
	Asthma	
	Coffee intake	
	Sugared drinks intake	
Liver steatosis	Male gender	0.38
	Body fat percent	
	Sedentariness	
FS30-lipid Full CVD risk	Hypertension	0.83
	APO-B	
	Male gender	
	Smoking	
	Age	
	APO-A	
	Body fat percent	
FS30-lipid major CVD risk	Hypertension	0.66
	Liver stiffness	
	Smoking	
FS30-BMI Full CVD risk	Hypertension	0.89
	Male gender	
	Smoking	
	Age	
	Liver stiffness	
	Diabetes	
	APO-B	
FS30-BMI major CVD risk	Hypertension	0.85
	Male gender	
	Smoking	
	Age	
	Diabetes	
	APO-B	

BMI, body mass index; CVD, cardiovascular disease; NS, not statistically significant. Importance is based on standardised beta coefficients in each model. Explanatory power is based on adjusted R-squared which ranged from 0 to 1, higher values indicating greater explanatory power of the predictors.

The risk of hard cardiovascular events obtained using FS30-Lipid was predicted by hypertension, FibroScan kPa, and smoking (in order of decreasing influence, as indicated by standardised beta coefficients) but not by other parameters, such as FibroScan CAP and FIB-4 (adjusted R-squared = 0.66).

The risk of full cardiovascular events obtained using FS30-BMI was predicted by hypertension, sex, BMI, smoking, age, FibroScan kPa, diabetes, and ApoB (in order of decreasing influence, as indicated by standardised beta coefficients) but not by other parameters, such as FibroScan CAP and FIB-4 (adjusted R-squared = 0.89).

**Figure 1:**
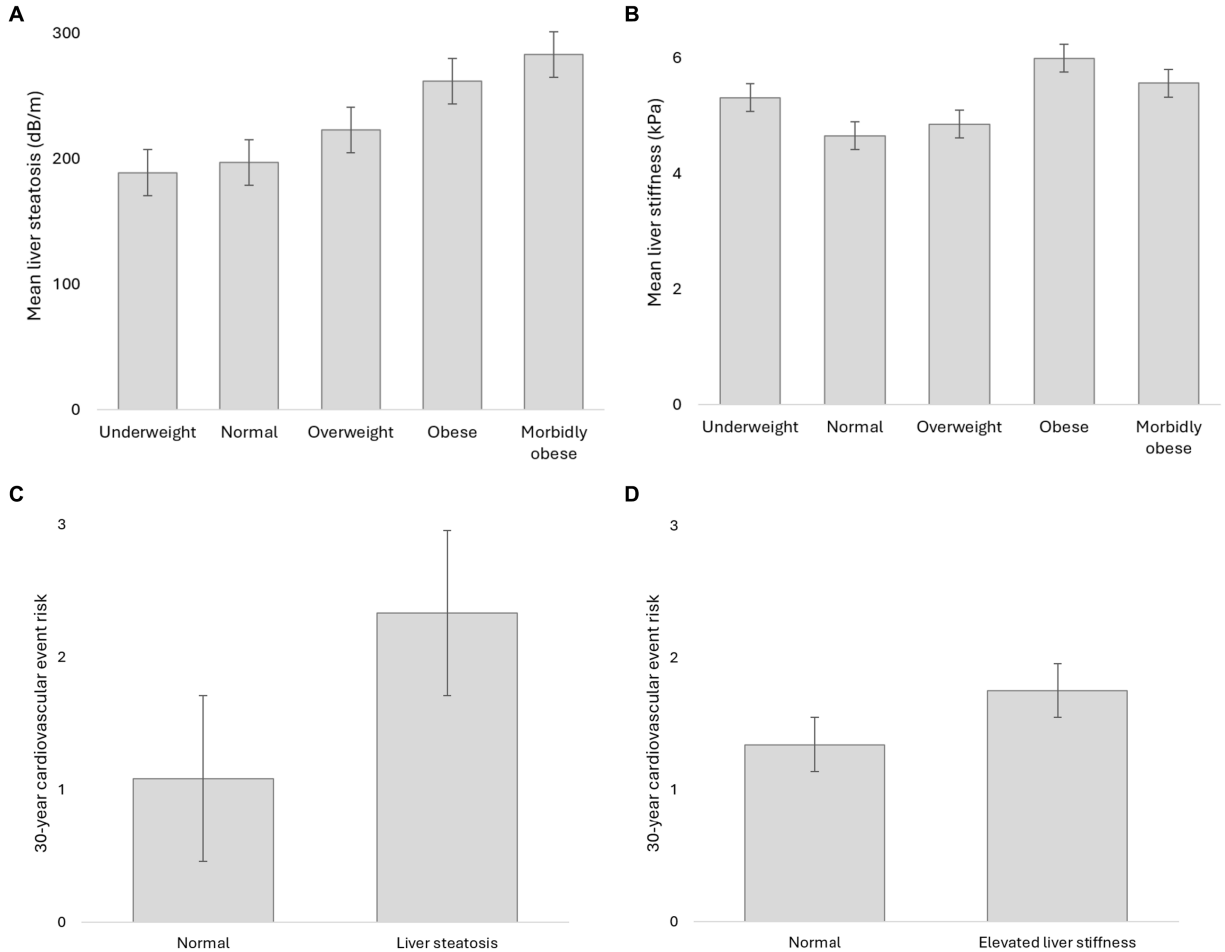
Hepatic and cardiovascular risk among young adults. **(A)** Liver steatosis. **(B)** Liver stiffness. **(C)** Liver steatosis; **(D)** Liver stiffness. 30-year cardiovascular risk estimates based on Framingham model for major (‘hard’) cardiovascular events based on BMI schema.

The risk of hard cardiovascular events obtained using FS30-BMI was predicted by hypertension, sex, BMI, smoking, age, diabetes, and ApoB (in order of decreasing influence, as indicated by standardised beta coefficients) but not by other parameters, such as MASLD measures, for example, CAP value or LSM in kPa, and FIB-4 (adjusted R-squared = 0.85; Figure 1).

## 4 Discussion

In this study of over 130 medical students, there was evidence of early liver damage and disease due to metabolic stress and obesity in almost 10% of subjects. There were two main findings of importance. Firstly, there was evidence of early onset of metabolic liver disease among young adults. Secondly, there were no reliable clinical or biomarker predictors of liver steatosis (apart from body fat and sedentariness). Other findings from our study include the following. Novel inflammatory biomarkers were not associated or predictive of liver damage. Fib-4 score seem to have diagnostic accuracy in this group of young adults.

To the best of our knowledge, this is the first study on the prevalence of MASLD and Framingham score among UAE medical students. This study was also the first that has established the association of CVD risk categories and MASLD in UAE. A study on CVD risk assessment in the gulf region has been performed in Saudi Arabia among university students and found the relationship of long-term cardiometabolic risk calculated via FS30-BMI to be strongly associated with central obesity indices such as visceral adiposity index and mid-arm muscular area in men and body mass index and waist circumference in women (13). Our study did not show any significant in these predictors of CVD risk but it did show that male participants were at disproportionately higher risk for developing full CVD on FS30-BMI with 40.4% (21/52) of males identified as compared to only 2.5% (2/79) of females and this difference was statistically significant with *p* value of <0.001. This 10-fold increased risk among males could be associated with factors such smoking, increased sugary intake, and consuming food from outside (Table 2), and this warrants further investigations.

**Figure 2:**
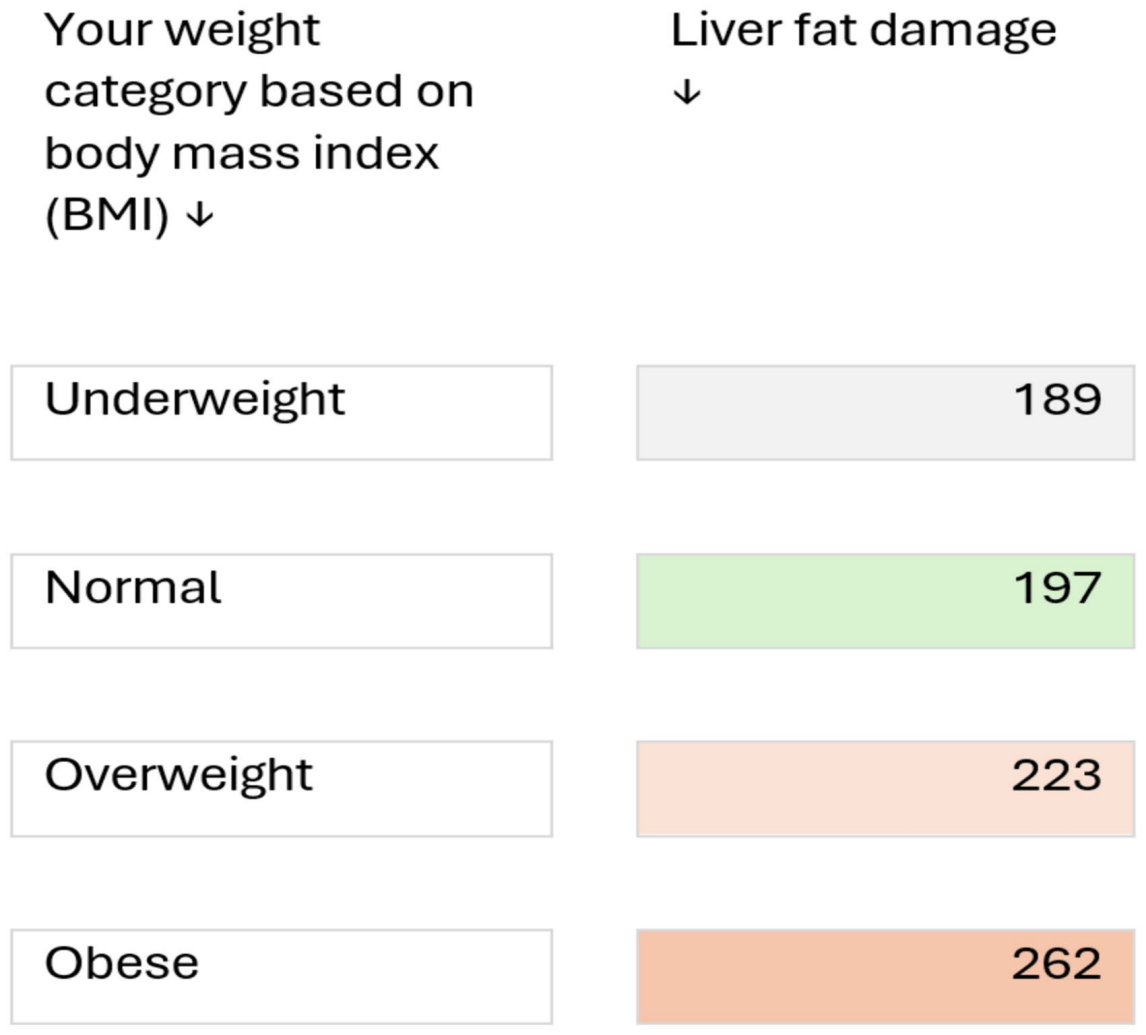
Clinical risk discussion tool for counselling healthy young adults at risk of metabolic liver disease (based on a study of 138 medical students, aged 17–24 years). Numbers indicate the level of liver fat damage (based on ultrasound measurements; values >236 dB/m indicate abnormal fat deposition in liver cells). Risk may be increased by other factors such as consumption of alcoholic beverages, certain medicines, genetic predisposition, and certain liver diseases, such as hepatitis B and C. Reducing weight may decrease the risk of metabolic liver disease.

In this study we assessed medical students for the evidence of liver stiffness and cardiovascular risk among UAE nationals using various diagnostic scores. We observed that theses scores are useful indicators of CVD risk and that liver stiffness is an important independent risk factor for CVD. The study showed evidence of early onset of liver stiffness and steatosis were associated with increased risk of CVD among young adults. The estimated prevalence of MASLD varies worldwide, ranging from 6 to 35% (19, 20). In a recent meta-analysis on the global epidemiology of MASLD, Younossi et al. (20) reported its prevalence to be highest (32%) in the Middle East. In our study, 10% students, who were apparently healthy, had liver stiffness, and 26.5% had steatosis (≥S1). In addition, we have demonstrated that male gender, body fat percent, sedentary life style were the main risk factors associated with hepatic fat accumulation. This is in contrast to study done in the Europe among Romanian medical students who had a prevalence of liver steatosis in only 17.4% while fibrosis (F2 and above) on FibroScan in only 3.1% of the participants while our medical students seems to have much higher rates of fibrosis and steatosis (21). A similar study in young Korean population showed prevalence of liver fibrosis among apparently healthy participants being 7% but it is still less than the one reported in our population (22). In light of the reported high frequency of liver steatosis in our population, we suggest a counselling tool (Figure 2) which visualises the level of liver fat damage in relation to BMI with a take home message that reducing weight may decrease the risk of MASLD.

Several previous studies have assessed the association between MASLD and CVD risk and have demonstrated that MASLD is an independent predictor of cardiovascular risk. A previous study argued that the mechanisms by which MASLD increases cardiovascular risk are very complex and involve multiple pathways, but that insulin resistance is the main determinant of MASLD pathogenesis (23). Another study reported evidence that MASLD is strongly associated with increased CVD risk, and that MASLD may not only be a marker, but also an early mediator, of atherosclerosis (24). Aside from insulin resistance, MASLD and atherosclerosis share unifying mechanisms involving pro-inflammatory, thrombogenic factors, and adipokines (25). Systemic diffusion of cytokines and chemokines due to hepatic necro-inflammation triggers vascular damage and coagulation system abnormalities; along with dysregulation of hepatokines can lead to MASLD (26). Expanded and inflamed visceral adipose tissue is the key mechanism linking MASLD with increased CVD risk with release of pro-inflammatory and pro-atherogenic factors, which may lead to the development of insulin resistance and atherogenic dyslipidemia, however due to genetic heterogenicity in MASLD pathophysiology CVD and insulin resistance is not always present (27).

Framingham Risk model has been used to predict 10-year CHD risk in adult UAE nationals without diabetes aged 30–79 without a baseline history of cardiovascular disease and diabetes (28). However, it has not been evaluated in young adults. In this study we correlated the liver steatosis/fibrosis and CVD risk among the young adults. Hypertension, male gender, smoking, age, were common predictors for Framingham Risk scoring.

## 5 Conclusion

MASLD with liver fibrosis and steatosis are both common in our young healthy asymptomatic population. This suggests that liver damage may appear at an early age; therefore, screening strategies and early intervention via lifestyle changes may be required in our young at-risk population. Our medical students seem to have higher rates of MASLD or liver steatosis when compared with similarly aged participants in other studies involving the West and far East regions. We also observed that the estimated CVD risk seems to be high in this group of participants, especially in young men. MASLD may pose cardiovascular risk beyond that conferred by traditional factors, such as dyslipidaemia, diabetes, and smoking. Healthcare providers managing individuals with MASLD should recognise this increased cardiovascular risk and should undertake early, aggressive risk-factor modification. Overall, the current body of evidence strongly suggests that MASLD is likely to be associated with increased CVD risk, and raises the possibility that MASLD may represent not only a marker but also an early mediator of atherosclerosis.

## 6 Limitations

Our study did not have sufficient numbers of participants for generalisability of the results to the broader population and further large-scale multi-centred prospective studies are required to confirm our findings. Our study used full CVD risk assessment using FS30-BMI and FS30-Lipid risk calculations and used a < 6% score as a cut-off for normal risk instead of classifying it into low or intermediate risk (6–12%), and high risk >12% scores. Also, the research subclinical biomarkers used in our study lacks established recommendations for use in clinical practice.

## Data Availability

The original contributions presented in the study are included in the article/supplementary material, further inquiries can be directed to the corresponding authors.

## Footnotes

^1^ https://www.framinghamheartstudy.org/fhs-risk-functions/

## Glossary

MASLD: metabolic dysfunction-associated steatotic liver disease
CVD: cardiovascular disease
BMI: body mass index
FS: Framingham risk score
LSM: liver stiffness measurements
CAP: controlled attenuation parameter
UAE: United Arab Emirates
ApoA: apolipoprotein A
ApoB: apolipoprotein B
PIGR: polymeric immunoglobulin receptor
IGHA2: immunoglobulin heavy constant alpha 2
HEP2: heparin cofactor 2
BF: body fat
ALT: alanine aminotransferase
AST: aspartate aminotransferase
SD: standard deviation
NFS: NAFLD fibrosis scores
FIB-4: fibrosis 4 index
HbA1c: glycosylated haemoglobin
